# Correlation between symptoms and course duration of upper aerodigestive tract cancer at early and advanced stages

**DOI:** 10.5935/1808-8694.20130125

**Published:** 2015-10-08

**Authors:** Francis Balduino Guimarães Santos, Jose Jacinto Branco Vasconcelos-Raposo, Maria do Carmo Tolentino Figueiredo

**Affiliations:** a MSc, UTAD, Portugal. (Head and Neck Surgeon, Professor at the UNIMONTES university medical program); b PhD. (Full Professor, UTAD - Portugal); c MSc and PhD from UNIFESP (Attending and Professor at UNIMONTES). State University of Montes Claros (UNIMONTES)

**Keywords:** clinical course, late diagnosis, neoplasms of the head and neck, primary prevention, signs and symptoms

## Abstract

There still are many clinical and biological aspects of the natural history of cancer of the upper aerodigestive tract to be unveiled; which in Brazil is a direct consequence of the failure of systematic prevention and early diagnosis campaigns.

**Objective:**

To analyze the signs and symptoms presented by patients with the disease at initial and advanced stages. Other variables such as disease duration, general and nutritional status were considered.

**Method:**

A historical cohort study with a cross-section involving 895 subjects with cancer of the upper aerodigestive tract.

**Results:**

Clinical findings were not statistically correlated with disease progression, nor with the disease in early stages, but it showed rapid disease development.

**Conclusion:**

The results suggest a disease of insidious onset in the early stages and fast course afterwards. The long disease duration - greater than three months, was associated with worsening in general and nutritional states of patients.

## INTRODUCTION

Head and neck cancer is the sixth most prevalent primary malignant neoplasm in the world, accounting for 5% of all cancers in men and 2.5 % in women^1^, being epidemiologically important in many countries worldwide, including Brazil2., 3..

The most affected anatomical site by head and neck cancer in the upper aerodigestive tract, distributed among the oral cavity (35%-40%), oropharynx (30%), larynx (25%) and hypopharynx (7%)^4^. The most prevalent histological type of cancer located in the upper aerodigestive tract is the Squamous Cell Carcinoma (SCC) in 90% of the cases, and its main etiological factors are tobacco smoking and alcoholic beverages drinking5., 6., 7., although infection by the human papilloma virus (HPV) has been responsible for at least 10% to 30% of oropharyngeal cancers^8^.

Clinical staging at the time of diagnosis is the most important prognostic factor affecting survival, but it is advanced around 75% of cases, especially in developing countries. As a result, about 40% to 60% of patients with SCC have locoregional recurrence and 20% to 30% progress with distant metastases^8^. After treatment, the overall mortality rate stands at around 40% to 60% in developed countries, with a global average around 46%^9^. The reasons described in the literature to justify this high incidence in advanced phases are distributed in a miscellany of situations: cultural and economic factors, poor social support, lifestyles, demographics and geographic factors, as well as difficulties in having access to healthcare10., 11., 12., 13.. There is no consensus in the literature regarding barriers to the early diagnosis of SCC.

Studies in Brazil show that the population is unaware of the risk factors associated with these cancers14., 15.. This is partially due to low levels of schooling and low household per capita income16., 17.. Another study suggests that the early diagnosis of SCC may be hampered by the fact that the initial lesions, with few symptoms, are not appreciated either by the individual or by healthcare workers^12^. They also suggest that these symptoms can be confused with other common diseases affecting the population. More so when these symptoms have never been presented to them as cancer harbingers18., 19.. Modern health thinking is based on the co-responsibility of the individual for his own health^20^.

In Brazil, there are selective campaign against oral cavity or laryngeal SCC and most of them have been highly ineffective, suggesting the need for further understanding the biological and epidemiological processes behind these types of cancer15., 16., 21., hence the finding of a carcinogenic lesion remains the most effective clinical intervention22., 23.. In terms of research, much emphasis has been given to the risk factors and their causal relationships, and less has been done regarding its natural clinical history.

Finally, the disease remains challenging because of its diagnoses in advanced stages, its high morbimortality^24^ and hence the negative impact on budgets. There are very few correlational studies between the various symptoms, clinical staging and disease course vis-à -vis the initial diagnosis, since it may help to understand human behavior in different regions of the world and outline an epidemiological profile with specific health actions concerning the population exposed to certain risk factors.

SCCs may be considered as a separate disorder in the four anatomical sites and traditionally focused on the specialist's view or be presented as a single disease25., 26.. In this approach, there is a strong increase in its incidence, taking into account similar epidemiological aspects, and there may be a convergence of combined health actions with cost reduction - vision focused on public health. With both approaches, the present study has the “overall objective” to describe the sociodemographic, behavioral, and clinical characteristics of the sample. The “secondary objectives” are to associate disease course with its staging in initial and advances stages, as well as establish the temporal relationship of the signs/symptoms with the patients' general and nutritional status at the time of diagnosis.

## METHOD

Historical Cohort Cross-Sectional Study - it follows the same characteristics of the longitudinal cohort study, however establishing a restricted time point (cut) to assess the elements studied, with no follow-up over time. For the study we selected 895 patients with SCC, between March 1996 and January 2009, seen at the head and neck surgery clinics located in Montes Claros/Minas Gerais - Brazil. We used standard patient charts from only one specialist. This study was approved (Resolution 131 - CEPEX/2008, Ethics Board under protocol # 005/2008) and did not receive funding for its execution. The variables used are described below.

Sociodemographic variables (gender, marital status, age group, place of residence and occupation) and lifestyle habits harmful to health, such as smoking and drinking alcoholic beverages, in addition to the following clinical variables: anatomical sites and subsites according to the International Classification of Diseases (ICD, 10^th^ ed., 2000)^27^ as follows: oral cavity, oropharynx, larynx and hypopharynx. The criteria used for staging followed the TNM system of the International Union Against Cancer (IUAC TNM, 6^th^ ed., 2002)^28^. Patients with diagnosis dates prior to 2002 had their staging updated under current TNM standards. Disease progression was considered as reported by the patient, between the dates of the first symptoms to the first visit to a specialist in head and neck surgery (short: ≤3 months; long: > 3 months). Nutritional status was assessed according to a subjective global assessment of the nutritional state29., 30. considering the patient's report of weight loss, current weight of the patient, the medical and physical characteristics presented by the patient or by the association of this information. The general health was assessed by the clinical classification from the Zubrod-Karnofsk^31^ Performance Status assessment.

We calculated the frequencies, mean values and odds ratios for possible associations between the variables investigated. We used the Statistical Package for Social Sciences (SPSS V. 19®). Initially, we performed a descriptive analysis of the data. Subsequently, due to the nature of the variables, we used the nonparametric tests, a bivariate analysis (x^2^ test, Mann-Whitney test) and multivariate data analysis (binary logistic regression). The significance level of 5% was retained for the statistical procedures performed (*p* = 0.05^*^).

## RESULTS

The conditions most frequently encountered were: males (84.8 %) in the ratio of 5.6 ♂: 1 ♀ Age ranged from 7 to 92 years, with a mean value of 59.6 years and incidence peaks between the 5^th^ (28.6 %) and 6^th^ (24.8 %) decades of life. We found a higher number of smokers/former smokers (91.5 %) and alcoholic beverages drinkers/former drinkers (86.4 %). As for skin color, the distribution was as follows: whites 227 (30.6 %), browns 417 (56.1 %) and blacks 99 (13.3%). Regarding marital status, married subjects represented 64.8 % (449), singles 17.3% (120), widow (er) 12 % (83), separated 4% (28) and 1.9% others (13).

The anatomical sites affected were distributed as follows: oral cavity (33.9%), larynx (28.8%), oropharynx (27.9%), and hypopharynx (9.4%) - Table 1. On this table one can also notice the most affected subsites. Considering all the anatomical sites, clinical staging upon diagnosis was advanced in 80.5% of patients. The anatomic site at which the disease was most advanced in stages III/IV was the hypopharynx with 96.3 % - Table 1.

**Table 1:** Distribution and frequency of patients with UADT SCC by anatomical site, according to staging (cTNM), general health, nutritional status and symptoms.

Clinical variables	Oral cav.	Oropharynx	Larynx	Hypopharynx n (%)	Total
Staging	n (%)	n (%)	n (%)	n (%)	%
0/I Initial	27 (15.3)	1 (0.4)	35 9 (13.9)	1 (1.2)	(0/I/II)
II	59 (19.6)	18 (7.4)	33 (13.1)	2 (1.8)	= 19.5
III Advanced	69 (27.1)	64 (26.4)	105 (41.7)	17 (20.7)	(III/IV)
IV	146 (32.7)	159 (65.7)	79 (31.3)	62 (75.6)	= 80.5
Total 1	301	242	252	82	877 (100%)
General health status					n (%)
90–100%	93 (31.8)	56 (23.7)	72 (30.8)	12 (15.0)	233 (27.7)
70–80%	162 (55.5)	130 (55.1)	133 (56.8)	46 (57.5)	471 (55.9)
50–60%	35 (12.0)	44 (18.6)	22 (9.4)	15 (18.8)	116 (13.8)
30–40%	0	4 (1.7)	3 (1.3)	7 (8.8)	14 (1.7)
0–20%	2 (0.7)	2 (0.8)	4 (1.7)	0	8 (0.9)
Total 2	292	236	234	80	842 (100)
Nutritional status					n (%)
Nourished	154 (53.5)	88 (37.3)	130 (55.8)	41 (51.3)	413 (49.3)
Emaciated	122 (42.4)	132 (55.9)	96 (41.2)	34 (42.5)	384 (45.9)
Malnourished	12 (4.4)	16 (6.8)	7 (3.0)	5 (6.3)	40 (4.8)
Total 3	288	236	233	80	837 (100)
Symptoms					N
Dysphonia	16 (5.9)	51 (22.4)	175 (77.8)	45 (57.0)	287
Dyspnea	2 (0.7)	21 (9.20)	69 (30.7)	17 (21.5)	109
Dysphagia	42 (15.5)	70 (30.7)	53 (23.6)	45 (57.0)	210
Pain	211 (77.9)	195 (85.0)	104 (46.6)	64 (81.0)	574
Emaciation report	28 (10.3)	25 (11.0)	15 (6.7)	15 (19.0)	83
Lesion report	135 (49.8)	34 (14.9)	7 (3.1)	0	176
Neck nodule report	45 (16.6)	68 (29.8)	28 (12.4)	22 (27.8)	163
Otalgia	162 (37.6)	108 (47.4)	45 (20.0)	32 (40.5)	347
Sensation foreign body	3 (1.1)	23 (10.1)	16 (7.1)	10 (12.7)	52
Pasty voice	14 (5.2)	17 (7.5)	1 (0.4)	1 (1.3)	33
Sorethroat	24 (8.9)	70 (30.7)	26 (11.6)	23 (29.1)	143
Coughing	2 (0.7)	6 (2.6)	14 (6.2)	3 (3.8)	25
Hawking	0	3 (1.3)	12 (5.3)	2 (2.5)	17
Halitosis	4 (1.1)	8 (3.5)	1 (0.4)	0	13
Trismus	11 (4.0)	6 (2.6)	0	1 (1.3)	18
Drooling	3 (1.1)	4 (3.1)	1 (0.4)	0	8
Bleeding	14 (5.2)	6 (2.6)	8 (3.6)	2 (2.5)	30

Considering the nutritional status, 52.1 % of the patients were emaciated or malnourished upon diagnosis and, as far as general condition is concerned, 55.9 % had mild to moderate impact - Table 1.

Symptoms were observed in two ways: separately, for each anatomical site and together - SCC being considered here as a single disease. In the former, and as expected, the most frequent symptoms were distributed in different ways for each anatomic site. The three main symptoms in order of frequency for the oral cavity were: pain, lesion reported by the patient and earache. The oropharynx had the following distribution of symptoms: pain, dysphagia and odynophagia. For the larynx, they were: dysphonia, pain and dyspnea, and for the hypopharynx: pain, dysphagia and dysphonia.

Otherwise, upon considering all anatomical sites together, some differences are noteworthy and the most prevalent symptom of the sample is represented by tumor pain (71.5 %); followed by reflex otalgia, dysphonia, dysphagia, reporting the lesion - the patient reporting a neck lump and dyspnea - Table 1.

After considering the differences in clinical behavior (signs and symptoms) of UADT SCC obtained by separate and joint evaluation of anatomical sites, one can notice their association with the initial and advanced staging.

Considering again the anatomic sites separately through a multivariate logistic regression between staging, anatomical site and symptoms, the results show statistically significant associations for advanced disease. They were statistically significant in the oral cavity, pain and injury reported by the patient; in the oropharynx, the reporting of a lump by the patient, otalgia and drooling; in the larynx: dysphonia, hawking and dyspnea; and in the hypopharynx: dysphonia, dysphagia, patient reporting a lump and a foreign body sensation - Table 2.

**Table 2:** The association of UADT SCC patient symptoms variables, according to anatomical site and stage, estimated by the multivariate logistic regression model. Odds Ratio (OR) with a 95% confidence interval (IC).

Anatomical site	Symptoms	OR (CI 95%)	*p*-value
Model 1:	Pain	1.64 (0.02–0.44)	0.047*
Oral cavity	Reporting the lesion	5.87 (1.01–2.68)	0.000*
	Reporting the lump	1.80 (1.22–2.63)	0.003*
Model 2:	Otalgia	1.61 (1.14–2.26)	0.006*
Oropharynx	Odinophagia	2.65 (1.78–3.96)	0.000*
	Drooling	4.94(1.35–18.05)	0.016
	Dysphonia	8.06(5.15–12.62)	0.000*
Larynx	Dyspnea	2.04 (1.19–3.50)	0.009*
	Hawking	4.53(1.15–17.85)	0.031
	Dysphonia	2.26 (1.37–3.71)	0.001*
Model 4:	Dysphagia	4.24 (2.59–6.95)	0.000*
Hypopharynx	Reporting of a lump	1.81 (1.03–3.17)	0.038*
	Foreign body	2.50 (1.14–5.45)	0.022*

* *p*-value < 0.05.

Considering now the anatomical sites together, through the distribution of symptoms according to staging, we obtained statistically significant associations between symptoms and advanced stage, these being: the report of the nodule, otalgia, dysphagia, dyspnea and the account of a lesion - Table 3.

**Table 3:** Distribution of variables: symptoms of patients with UADT SCC, according to staging. Odds Ratio (OR - odds ratio) with a confidence interval of 95% (CI).

		Staging		Total	OR	
Symptom		Advanced III/IV	Initial 0/I/II			*P*
		n (%)	n (%)	n (100%)	(IC-95%)	
Dysphagia	Yes	195 (30.6)	13 (8.6)	208	2.6 (1.4–5.0)	0.004*
	No	442 (69.4)	138 (91.4)	580	1.0	
Dysphonia	Yes	218 (34.2)	60 (39.7)	278	0.69 (0.4–0.2)	0.18
	No	419 (65.8)	91 (60.3)	510	1.0	
Dyspnea	Yes	91 (86.7)	14 (9.3)	105	2.2 (1.1–4.3)	0.027*
	No	546 (85.7)	137 (90.7)	683	1.0	
Pain	Yes	492 (77.2)	76 (50.3)	568	1.62 (1.0–0.6)	0.052
	No	145 (22.8)	75 (49.7)	220	1.0	
Emaciation report	Yes	77 (12.1)	4 (2.6)	81	2.8 (1.0–8.2)	0.059
	No	560 (87.9)	147 (97.4)	707	1.0	
Lesion report	Yes	120 (18.8)	52 (34.4)	172	2.15 (0.3–0.9)	0.021*
	No	517 (81.2)	99 (65.6)	616	1.0	
Lump report	Yes	156 (24.5)	6 (4.0)	162	7.6 (3.2–18.1)	0.000*
	No	481 (75.5)	145 (96)	626	1.0	
Otalgia	Yes	266 (41.8)	21 (13.9)	287	2.6 (1.5–4.6)	0.001*
	No	371 (58.2)	130 (86.1)	501	1.0	
Foreign body	Yes	41 (6.4)	9 (6)	50	0.95 (0.4–2.2)	0.91
	No	569 (93.6)	142 (94.0)	738	1.0	
Pasty voice	Yes	31 (4.9)	2 (1.3)	33	1.4 (0.3–6.6)	0.69
	No	606 (95.1)	149 (98.7)	755	1.0	
Odinophagia	Yes	134 (21.0)	8 (5.3)	142	2.1 (0.9–4.7)	0.087
	No	503 (79.0)	143 (94.7)	649	1.0	
Coughing	Yes	19 (3.0)	5 (3.3)	24	0.81 (0.3–2.6)	0.72
	No	618 (97)	146 (96.7)	764	1.0	
Hawking	Yes	8 (1.3)	7 (4.6)	15	0.31 (0.9–1.0)	57
	No	773 (98.1)	144 (95.4)	917	1.0	
Bleeding	Yes	19 (3.0)	10 (6.6)	29	0.55 (0.2–1.3)	0.18
	No	618 (97.0)	141 (93.4)	759	1.0	

* *p*-value < 0.05.

We found statistically significant associations between staging in advanced stages of the disease with the general and nutritional status variables - Table 4.

**Table 4:** Distribution of variables: general and nutritional status of patients with UADT SCC as per staging and binary logistic regression analysis. Odds Ratio (OR) with a confidence interval of 95% (CI).

		Staging		Total	OR	
Variable		Advanced	Initial			*P*
		n (%)	n (%)	n	(CI-95%)	
General health	Compromized	129 (19.6)	5 (3.1)	134	7.7 (3.0–19.0)	0.000*
	Preserved	530 (80.4)	157 (96.9)	687	1.0	
Nutritional status	Emaciated or malnourished	388 (58.9)	41 (25.2)	429	4.2 (2.9–6.3)	0.000*
	Nourished	271 (41.1)	122 (74.8)	393	1.0	

* *p*-value < 0.05.

We found no association between disease course and its staging. There was no statistically significant difference between disease course and the initial or advanced stages. Unlike hypothesized (H_1_), the disease course did not correlate with staging; however, the long disease course (over three months) was correlated with worsening nutritional states and general health of the patients - Table 5. The time of complaints reported by patients (n = 778) ranged from 1 to 60 months (30 to 1,800 days), with an average of 150 days (SD = 235.44).

**Table 5:** Descriptive measure (mean rank) of disease course according to staging, patient's general condition and nutritional status, and the Mann-Whitney test.

Course (months)	Staging	General health	Nutritional status
	n (mean rank)	n (mean rank)	n (mean rank)
≤ 3 months	251 (364.95)	250 (361.00)	250 (350.23)
> 3 months	493 (376.34)	500 (382.75)	501 (388.86)
*P*	315	0.043*	0.008^**^

* *p*-value < 0.05.

One can observe the variables that correlate with worsening patient nutritional status, such as dyspnea, dysphagia, weight loss reported by the patient, otalgia and trismus. Similarly, the nutritional status variable correlated with general health worsening. Now, ‘dysphagia and dyspnoea’ were statistically significant vis-à -vis the worsening in their general health - Table 6.

**Table 6:** Association between the general health and nutritional status of UADT SCC patients with symptoms*; estimated by the binary logistic regression model. Odds Ratio (OR) with a 95% confidence interval (CI).

General health			OR (CI 95%)	*p*
Symptoms	Dyspnea	Yes	2.33 (1.39–3.88)	0.001*
		No	1.0	
	Dysphagia	Yes	1.93 (1.26–2.95)	0.003*
		No	1.0	
Nutritional status				
Symptoms	Dyspnea	Yes	2.41 (1.52–3.82)	0.000*
		No	1.0	
	Dysphagia	Yes	2.66 (1.85–3.84)	0.000*
		No	1.0	
	R.Emanc.	Yes	3.24 (1.82–5.77)	0.000*
		No	1.0	
	Otalgia	Yes	1.63 (1.19–2.25)	0.003*
		No	1.0	
	Trismus	Yes	3.74 (1.19–11.80)	0.025*
		No	1.0	

* We considered here only those variables with statistically significant results.

* *p*-value < 0.05.

## DISCUSSION

In this study, the epidemiological profile of the UADT SCC patients was similar to findings published in the literature32., 33., 34.. The significant findings regarding gender 5.6 ♂: 1 ♀, are partly justified by sociocultural factors; men being more exposed to carcinogenic agents for the development of UADT SCC (risk factors: tobacco and alcohol). The advanced stage found in 80.5% of patients did not differ from the literature33., 34. but confirms the seriousness regarding the epidemiology of UADT SCC.

Pain, in its various forms, was the most prevalent general symptom, affecting 71.5 % of patients with UADT SCC, possibly due to the high number of patients with advanced disease, similar to that of another study^35^. It is known that pain increases the degree of suffering of patients, with significant reduction in hours of sleep, leading to drops in productive capacity, absenteeism and money spent with medications, which ended in a *Via Crucis* for social support and medical care. This fact shows the importance of pain as a recognition factor of the state of sickness of UADT SCC patients^19^, but this, unfortunately, already predicts advanced disease. Such as pain, many signs and symptoms are associated with advanced disease in a statistically significant fashion, which does not favor prognosis.

The UADT SCC, which was evaluated by two approaches, as different diseases or as a single disease, presented somewhat similar information. The former had heterogeneous symptoms, which is unevenly distributed among the four anatomical sites, but did not show any pathognomonic sign or symptom to either location. Although the anatomical sites are distinct and have different functions together, their anatomical proximity can make large tumors involve more than one anatomical site or subsite simultaneously, thus producing mixed symptoms. The symptoms listed here were associated with advanced disease. The UADT SCC evaluated as a single disease had symptoms associated with advanced disease such as reporting of the lump, dysphagia, reports of a lesion and dyspnea.

It is difficult to understand why UADT SCC is not diagnosed at an initial stage, but the various symptoms were mostly associated to an advanced stage. Possibly, the initial symptoms are subtle, disregarded by the patient and the disease evolves progressively and rapidly to the advanced stage, not yielding to an initial diagnosis in proper time^19^.

However, the population has no knowledge or clinical conditions to identify initial or precancerous UADT SCC lesions12., 19., 23., a finding essential to prevention. The active search for precursor UADT SCC lesions (screening) should be reconsidered for individuals with risk factors for its development (over 45 years old, smoker and/or drinker), as already observed in other studies with favorable results35., 36., 37.. There is a range of medical equipment currently available to perform imaging of the upper aerodigestive tract (laryngoscopy), which can be offered to the community by the public healthcare system; however, at a cost to be assessed. Primary and secondary care require investments in physical infrastructure and technology for proper action in the prevention and early diagnosis of UADT SCC, as it was done for cervix cancer^38^.

Some symptoms were significantly associated with the physical decline of patients, such as dyspnea, dysphagia and weight loss. Individuals with mechanical constraints for breathing and/or feeding, due to tumor invasion, have their nutritional status compromised^39^. These symptoms may be the ones most associated with the wasting state of the disease and can have an unfavorable impact on treatment and prognosis.

The disease course between the first signs and symptoms until the patient's motivation to seek medical care was long in this series (patient's delay), around 150 days, but also comparable to results published in the literature^40^. The long disease course (> three months) correlated with worsening nutritional and general health status, similarly to that found by other studies, require these patients to receive greater attention in terms of clinical ad nutritional support41., 42..

## CONCLUSION

Among the results, two were really striking when considering UADT SCC as a single disease: (1) there were no statistically significant signs and symptoms of early UADT SCC, and (2) there was no correlation between the disease course and its staging (patient's delay). This proved, and as the most important conclusion, that when the symptoms become noticeable to patients, they are already an indication of an abnormality, in fact already foreshadowing advanced disease, suggesting rapid evolution of early disease stage to more advanced stages.

The long course of UADT SCC - more than three months, can be regarded as a predictor of worsening in the patient's general and nutritional status. Similarly and specifically, symptoms of “dyspnea and dysphagia” joined the overall status of these patients.

## CONFLICTS OF INTEREST

The authors declare no conflicts of interest.
